# Evaluating the coupling coordination between Healthy Beijing initiative and economic development

**DOI:** 10.3389/fpubh.2025.1674436

**Published:** 2025-11-26

**Authors:** Xi Wang, Yueying Cui, Jiu Cheng, Mingming Gao, Yifei Wang, Ruihua Feng

**Affiliations:** 1 Institute of Medical Information / Medical Library, Chinese Academy of Medical Sciences and Peking Union Medical College, Beijing, China; 2 Union Hospital, Tongji Medical College, Huazhong University of Science and Technology, Wuhan, China

**Keywords:** Healthy Beijing initiative, coupling coordination, Healthy China initiative, economic development, evaluating

## Abstract

**Background and objective:**

Health and economic development are pivotal elements underpinning societal progress, with intricate mutual influences. Under China’s “Healthy China” Initiative, the “Healthy Beijing Initiative” plays a crucial role in promoting coordinated health-economic development in the capital. This study aims to evaluate the coupling coordination between the Healthy Beijing Initiative and economic development in Beijing from 2020 to 2023, addressing imbalances and spatial disparities in their interactive development.

**Methods:**

The study employed the Global Entropy Value Method for dynamic indicator weighting and the Coupling Coordination Degree (CCD) model to measure system interactions. Municipal and district-level data were used: health indicators were sourced from the Healthy Beijing Initiative Monitoring Report, and socioeconomic metrics from the Beijing Statistical Yearbook. ArcGIS was applied to visualize spatial variations in coupling coordination levels across 16 districts, with quantitative disparity indicators (coefficient of variation [CV], extreme ratio) used to analyze regional gaps.

**Results:**

The overall coupling coordination degree (D) showed an upward trend, transitioning from “basic coordination” (D = 0.68) in 2020 to “intermediate coordination” (D = 0.714) in 2023, driven by synergies between health infrastructure investments and economic policies. Subsystem analysis revealed disparities: health status (D = 0.748) and services (D = 0.726) maintained sustained “intermediate coordination,” while health security (D = 0.683) and environment (D = 0.665) lagged due to volatile resource allocation and persistent environmental challenges. Spatially, core urban districts (e.g., Xicheng, D > 0.9) achieved “high-quality coordination,” contrasting with exurban areas (e.g., Pinggu, D < 0.5) plagued by infrastructure gaps and health-economic decoupling.

**Conclusion:**

Targeted policies are required to address subsystem imbalances (especially in health security and environmental governance) and spatial inequities. This study provides empirical evidence for integrated health-economic planning in megacities. Limitations include a 4-year data span and reliance on quantitative metrics; future research should extend the study period and integrate qualitative analyses to deepen insights.

## 1 Introduction

In this day and age, health and economic development stand as two pivotal elements underpinning the progress of humanity society. Their interconnection has become increasingly intricate, with each exerting a profound and far - reaching influence on the other (1). As individuals’ aspirations for a high - quality life escalate, health has emerged as one of the crucial barometers for gauging the level of social development (2). Simultaneously, the sustained growth of the economy furnishes a robust material foundation and a powerful driving force for the advancement of the health sector (3).

From a global vantage point, nations across the globe are vigorously delving into strategies to achieve the harmonious co-development of health and the economy. Many countries have established sophisticated medical and healthcare systems. Their overarching goal is to elevate the health standards of their citizens, thereby fostering stable economic growth (4). For instance, several Nordic countries have implemented universal health - coverage initiatives, ensuring that every citizen has access to convenient and accessible medical services (5, 6). This not only bolsters the overall health of the population but also furnishes a reliable human - resource safeguard for economic development. In contrast, although confronted with numerous hurdles, developing countries are also ramping up their investment in the health domain, striving to enhance the health status of their individuals in a bid to realize sustainable economic growth (7).

In China, the nexus between health and economic development has garnered significant attention. Against the backdrop of rapid economic growth and technological progress (8), economic expansion has not always translated to improved population welfare; instead, it has brought serious public health challenges—such as a looming burden of non-communicable diseases that will strain future health systems and constrain economic growth (9). In response, the Chinese government has propounded the “Healthy China” Initiative, which aims to provide all-encompassing, life-cycle health protection for the populace and lay a health-based foundation for socioeconomic development.

Under the “Healthy China” framework, recent domestic studies have advanced our understanding of health-economy interactions, yet critical gaps remain. Sun et al. (10) highlighted that China’s “Year of Weight Management” initiative (2024–2027) and the 36th Patriotic Health Month theme (“Healthy Towns - Healthy Weight”) have underscored obesity control as a core pillar of “Healthy China,” but these efforts lack spatial targeting for megacities. For “Healthy Beijing” specifically, Dai et al. (11) analyzed health resource allocation before and after the 13th Five-Year Plan for Healthy Beijing (2016–2020), finding that while health human resources grew at an annual rate of 24.6%, inter-regional disparities persisted—over 70% of health resource inequity stemmed from gaps between functional zones. Cheng et al. (12) further quantified the “Healthy Beijing” action index (2020–2022) using the global entropy method, identifying “per 1,000 residents practicing (assistant) physicians” and “government health expenditure ratio” as key obstacles to balanced health-economy development, yet their study did not establish a direct link between these obstacles and economic growth gradients. Additionally, Cao et al. (13) explored the association between adolescent health-related physical fitness and depressive symptoms under “Healthy China,” emphasizing the need for mental health integration into health-economy coordination, but this research focused on individual health outcomes rather than systemic coupling. These studies collectively indicate that domestic research on “Healthy China” and “Healthy Beijing” has strengthened temporal dynamics and regional specificity, but lacks in-depth analysis of the quantitative interaction mechanism between health initiatives and economic development—especially for megacities like Beijing.

As the capital’s concrete practice under “Healthy China,” the construction of “Healthy Beijing” encompasses multiple dimensions: enhancing residents’ health levels, promoting healthy lifestyles, refining the health service system, and strengthening health security. Through policy measures such as bolstering medical and healthcare infrastructure, improving medical service quality, and advancing health-related public education, “Healthy Beijing” has yielded remarkable outcomes—residents’ health awareness has risen, healthy habits have permeated society, and the accessibility and quality of medical services have improved. These changes not only enrich residents’ quality of life but also inject new impetus into economic development. Conversely, economic development profoundly influences “Healthy Beijing” construction: it provides ample financial resources for medical and healthcare investment, and rising residents’ income drives diverse, individualized health demands—fueling the growth of health-related industries as new economic growth areas.

However, issues have emerged in the interaction between “Healthy Beijing” and economic development: uneven allocation of health resources, immature development of the health industry, incomplete collaboration mechanisms between health and economic development, and underutilized mutually reinforcing effects. To address these issues, a quantitative tool is needed to assess the interaction and coordination level between the two systems—and the Coupling Coordination Degree (CCD) model is precisely such a tool. Yet, the application of the CCD model in public health-economic development remains underdeveloped and fragmented. The application of the coupling coordination degree model abroad focuses on fields such as ecological environment (14), land use (15), energy and resources regional development (16). Research in public health and economics is relatively scarce, and relevant studies are mostly carried out by constructing evaluation index systems and combining methods such as the entropy weight method. Domestically, most CCD studies have focused on ecological environment-economic systems (17) or urbanization-resource systems (18), with limited application to public health (4, 9). In rare public health-related CCD studies, Bian et al. (19) proposed a game theory-coupled weight method to optimize CCD index weights, addressing the subjectivity of traditional entropy weight methods, but their research focused on water resource allocation rather than health-economic interactions (7). Chen et al. (20) developed an improved CCD model based on game theory to evaluate mineral resource exploitation-economic-environment coupling, demonstrating the potential of multi-subsystem weight optimization, yet it did not involve public health indicators (8). These gaps indicate that the CCD model’s application in public health-economic development is characterized by three limitations: (1) subjective weight assignment (relying solely on entropy weight or AHP); and (2) lack of spatial–temporal dynamic analysis (ignoring regional disparities and long-term trends).

Against this backdrop, this study innovates the CCD model application in two key aspects, addressing the aforementioned limitations and establishing differentiated advantages:

(1) Optimizing weight assignment with global entropy-weighted CCD: While traditional CCD studies (19, 20) use single entropy weight or subjective weights, this study adopts the global entropy method (12) to dynamically assign weights to health and economic indicators across time and space, reducing subjective bias. This method is more robust than Bian‘s et al. (21) game theory weight method in handling multi-year panel data, as it integrates temporal variability into weight calculation.
(2) Integrating spatial analysis to capture regional disparities: Unlike most CCD studies that lack spatial dimension (22), this study combines ArcGIS spatial visualization with CCD results to analyze the spatial gradient of health-economic coupling in Beijing’s 16 districts. This addresses the limitation of Gan’s et al. urban-population-industry CCD model, which ignores intra-urban spatial heterogeneity (20).

To fill the existing research gaps and systematically evaluate Beijing’s health-economic coupling coordination, this study focuses on three key research questions:

(1) What dynamic trends does the overall coupling coordination between the “Healthy Beijing” Initiative and economic development show from 2020 to 2023, and is its upward trajectory statistically significant?
(2) Do the five health subsystems (health status, lifestyle, services, security, environment) have heterogeneous coordination levels with economic development, and which are the main bottlenecks?
(3) What spatial patterns exist in coordination across Beijing’s 16 districts, and what core factors drive “core-suburban-exurban” disparities?

Conducting in-depth research on the coupling and coordination relationship between “Healthy Beijing” construction and economic development is thus of significant practical importance for identifying problems, resolving conflicts, and achieving positive interaction and coordinated development between the two.

## 2 Materials and methods

### 2.1 Data resources

This study utilized data spanning 2020–2023 from both municipal and district levels in Beijing, encompassing 16 administrative districts. Health-related indicators were derived from the Healthy Beijing Initiative Monitoring Report, which systematically tracks progress in public health infrastructure, service delivery, and population health outcomes. Socioeconomic development metrics, including GDP per capita, employment rates, and environmental indices, were sourced from the authoritative Beijing Statistical Yearbook.

### 2.2 Data processing and analytical tools

Raw datasets were harmonized using Microsoft Excel (v2019) to ensure temporal and spatial consistency. Advanced analytics were conducted through SPSSAU and SPSSPRO for correlation matrices and coupling coordination degree calculation, adhering to rigorous quality control protocols. Regional variations in coupling coordination degrees were mapped through ArcGIS version 10.8.1. Spatial analysis parameters were configured as follows:

(1) Spatial weight matrix: Adopted the Queen adjacency matrix to define the spatial connection between districts (i.e., two districts are considered adjacent if they share a common boundary or vertex), which is suitable for Beijing’s administrative division characteristics.
(2) Standard deviational ellipse (SDE): Set the confidence level to 95% (default for spatial distribution analysis), with the long axis representing the main direction of CCD spatial variation and the short axis representing the secondary direction.
(3) Spatial autocorrelation test: Calculated Global Moran’s I index (using row-standardized weights) to verify the spatial agglomeration of CCD values, with the test statistic Z-score used to judge significance (Z > 1.96 indicates significant agglomeration at the 5% level).

### 2.3 Construction of index system

To comprehensively reflect the coordination relationship between health and economic development, this study constructs the index system on the basis of Dahlgren and Whitehead’s Social Determinants of Health (SDOH) ecological model (19), which emphasizes “modifiability” as the core principle for selecting health-related indicators. Referring to Cheng et al. (19) and the monitoring indicators of the Healthy Beijing Initiative (2020–2030), the health system indicators are divided into five dimensions (health status, healthy lifestyle, health services, health security, healthy environment). Although considered an important predictor of health, genetics was excluded from the model because this factor is currently largely unmodifiable (19). The detailed indicators of each system are shown in Table 1. Based on the relevant content of the National Bureau of Statistics of China (20) and the research of John F. Shroder et al. (23), the indicators of social and economic system are sorted out, including the per capita disposable income of residents (yuan), per capita GDP (yuan), the proportion of education expenditure in the general public budget expenditure, and the registered urban unemployment rate (%).

**Table 1:** Health index system for healthy Beijing initiative.

Dimension	Indicator	Attribute
Health status	Life expectancy at birth (years)	Positive
	Infant mortality rate (per 1,000 live births)	Negative
	Under-5 mortality rate (per 1,000 live births)	Negative
	Maternal mortality ratio (per 100,000 population)	Negative
Healthy lifestyle	Health literacy rate (%)	Positive
	Per capita sports facilities area (m^2^)	Positive
	Smoking prevalence among population aged ≥15 years (%)	Negative
	Excellent/good rate of National Student Physical Fitness Standard compliance (%)	Positive
	Myopia prevalence among children and adolescents (%)	Negative
Health services	Standardized management rate of severe mental disorders (%)	Positive
	Prenatal screening coverage (%)	Positive
	Coverage rate of basic rehabilitation and assistive devices for disabled persons (%)	Positive
	Proportion of public general hospitals (Grade II+) with geriatrics departments (%)	Positive
	Cardiovascular disease mortality rate (per 100,000 population)	Negative
	Chronic respiratory disease mortality rate (≤70 years, per 100,000)	Negative
	Proportion of TCM non-pharmacological therapies available in community health centers (%)	Positive
	Incidence rate of Class A/B notifiable infectious diseases (per 100,000)	Negative
	Number of licensed (assistant) physicians per 1,000 population	Positive
Health security	Reimbursement ratio of inpatient expenses under urban–rural resident medical insurance (%)	Positive
	Government health expenditure as percentage of fiscal expenditure (%)	Positive
Health environment	Percentage of days with good air quality (%)	Positive
	Per capita public green space area (m^2^)	Positive
	Compliance rate of drinking water quality standards (%)	Positive

### 2.4 Statistical methods

#### 2.4.1 Global entropy value method dynamic evaluation model method

The five systems scores and social and economic system scores were measured by the global entropy weight method via the following steps (18, 24, 25):

(1) Indicator standardization. To eliminate the influence of different measurement units and indicator directions, the min-max standardization method was used (range [0.1, 1.0] to avoid zero values affecting subsequent calculations):

For positive indicators (higher values indicate better performance):


$${\left(x_{\mathrm{ij}}^{t}\right)}^{'}=\frac{x_{\mathrm{ij}}^{t}-x_{j\min}}{x_{j\max}-x_{j\min}}\times 0.9+0.1$$

For negative indicators (lower values indicate better performance):


$${\left(x_{\mathrm{ij}}^{t}\right)}^{'}=\frac{x_{j\max}-x_{\mathrm{ij}}^{t}}{x_{j\max}-x_{j\min}}\times 0.9+0.1$$

In the formula, $x_{\mathrm{ij}}^{t}$ represents the value of the *j*-th indicator of the *i*-th region in the *t*-th year.

(2) Indicator weight calculation. The weight $w_{j}$ of the indicators, and the procedures are as follows:

Calculate the proportion of the indicator values: $\;f_{\mathrm{ij}}^{t}=\frac{(x_{\mathrm{ij}}^{t})\prime }{\overset{t=1}{\underset{T}{\Sigma }}\overset{i=1}{\underset{m}{\Sigma }}(x_{\mathrm{ij}}^{t})\prime }$ .

Calculate the information entropy of the *j*-th indicator based on the definition of information entropy in information theory: $\;e_{j}=-K\overset{t=1}{\underset{T}{\Sigma }}\overset{i=1}{\underset{m}{\Sigma }}f_{\mathrm{ij}}^{t}\ln f_{\mathrm{ij}}^{t}$ , In the formula, $\;K=\frac{1}{\ln\mathrm{mT}}$ .

Finally, calculate the weights of various indicators: $\;w_{j}=\frac{1-e_{j}}{\overset{j=1}{\underset{n}{\Sigma }}(1-e_{j})}$ . In the formula, $\;1-e_{j}$ is the deviation coefficient.

(3) Comprehensive index calculation. The comprehensive score $S_{i}$ is measured by calculating the weighted sum of the dimensionless indicators $(x_{\mathrm{ij}}^{t})\prime$ and their respective weights $w_{j}$ . The specific calculation formula is as follows: $\;S_{i}=\overset{j=1}{\underset{n}{\Sigma }}w_{j}(x_{\mathrm{ij}}^{t})\prime$ .

#### 2.4.2 Coupling coordination degree model

The coupling coordination degree model can better evaluate and analyze the coordinated development between two or more systems. The model calculation is simpler, and the results are more intuitive. Therefore, in this study, the scores of Beijing City and its 16 districts in the comprehensive health system, five health subsystem, as well as the social and economic development system, which are calculated by the entropy weight method, are used. The coupling coordination degree model is employed to measure the coordination degree between the health situation in Beijing and the social and economic development status (26–29).

(1) Calculate the coupling degree

The coupling degree C reflects the degree of correlation between systems, and the calculation formula is:


$$C=2\times \sqrt{\frac{U_{1}\times U_{2}}{{\left(U_{1}+U_{2}\right)}^{2}}}$$

Where U_1_ is the comprehensive index of the health system, U_2_ is the comprehensive index of the economic system; C∈[0,1], and C closer to 1 indicates stronger interdependence.

(2) Calculate the coordination coefficient

The coordination index T reflects the degree of coordinated development of the system, and the calculation formula is:


$$T=\beta_{1}\times U_{1}+\beta_{2}\times U_{2}$$

*β* represents the weight value of the system, and U is the system data. The weights of the two systems are the same. In this case, the value of *β* is all 1/2, and 2 is the number of systems.

(3) Calculate the coupling coordination degree

The coupling coordination degree D takes into account both the coupling degree and the coordination degree, and the calculation formula is:


$$D=\sqrt{C\times T}$$

The value of D cannot be less than 0. For this reason, generally, it is expected that both the value of C and the value of T are greater than 0 to ensure the normal calculation of the value of D (Table 2).

**Table 2:** Classification of the CCD (36).

Coupling coordination degree (D)	Classes
(0.0 ~ 0.1)	Extreme disorder
(0.1 ~ 0.2)	Serious disorder
(0.2 ~ 0.3)	Moderate disorder
(0.3 ~ 0.4)	Mild disorder
(0.4 ~ 0.5)	Near disorder
(0.5 ~ 0.6)	Barely coordinated
(0.6 ~ 0.7)	Primary coordination
(0.7 ~ 0.8)	Intermediate coordination
(0.8 ~ 0.9)	Well coordination
(0.9 ~ 1.0)	High-quality coordination

## 3 Results

### 3.1 Analysis of the coupling coordination degree of the overall situation in Beijing

The coupling coordination dynamics between the health system and economic development in Beijing during 2020–2023 exhibit distinct evolutionary characteristics (Table 3). The coupling degree (C) maintained an extremely high level (mean 0.988 ± 0.004) with minor fluctuations. Specifically, C-values were 0.992 in 2020, 0.985 in 2021, 0.985 in 2022, and 0.992 in 2023, showing no obvious decline during 2021–2022 despite the impact of COVID-19, which implies a strong and stable coupling relationship between the two systems throughout this period.

**Table 3:** Coupling coordination degree of the overall situation in Beijing.

Year	C	T	D
2020	0.992	0.466	0.680
2021	0.985	0.495	0.698
2022	0.985	0.518	0.714
2023	0.992479	0.514	0.714

In contrast, the comprehensive development index (T) for these two newly calculated indices demonstrated growth. It increased from 0.466 in 2020 to 0.514 in 2023, with a cumulative increase rate of approximately 10.4%. Its annual growth rate also showed some fluctuations, with the rate from 2020 to 2021 being higher than that from 2022 to 2023.

**Figure 1:**
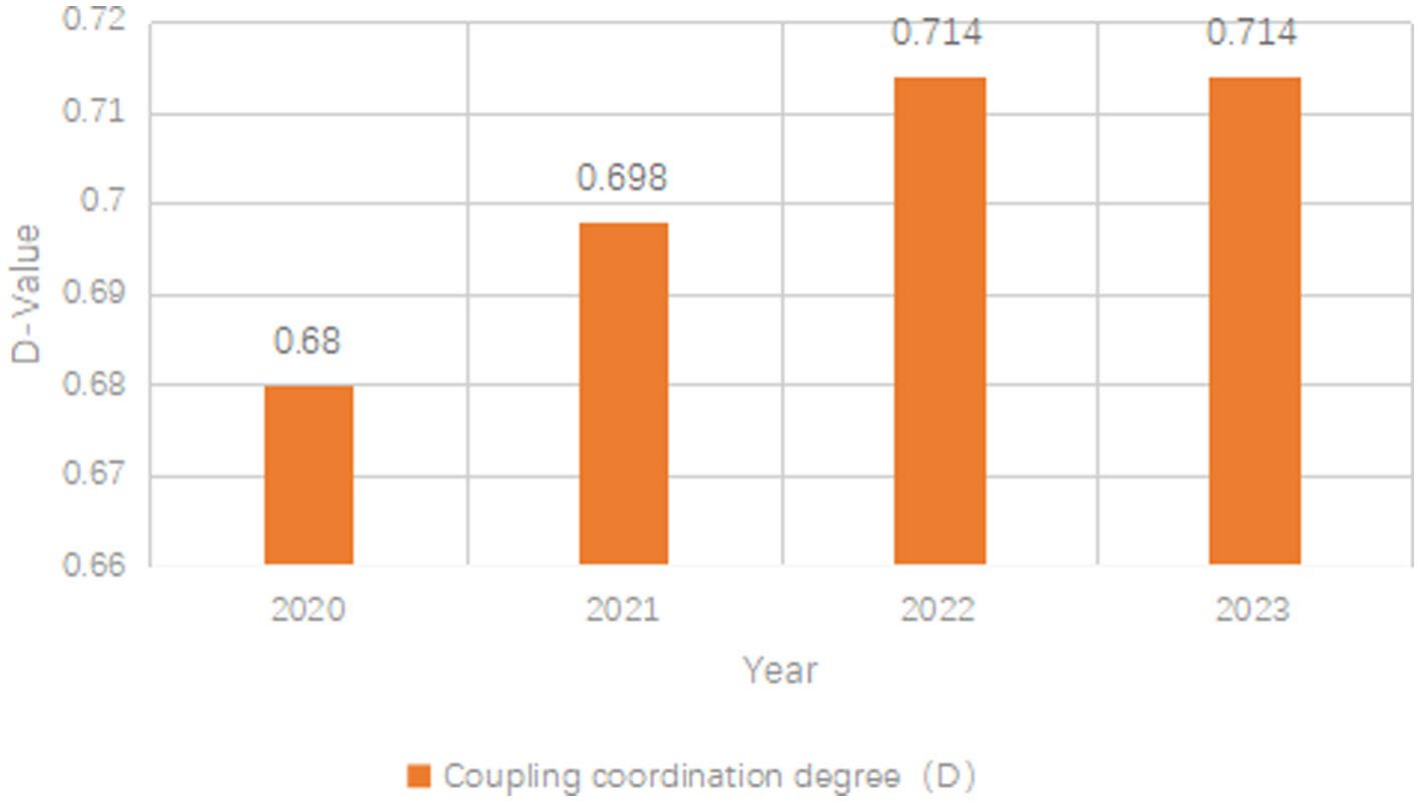
The results of the coupling and coordination degree between the health status and social and economic development in Beijing.

Concurrently, the coordination level (D) also showed a steady upward trend. It increased from 0.680 in 2020 to 0.714 in 2023, with an overall increase of about 5.1%. The successive annual increments were approximately 0.019 (2021), 0.016 (2022), and 0.004 (2023), reflecting enhanced systemic stability. A linear regression analysis with year as the predictor was conducted to verify the significance of the upward trend in D. The results showed that the slope of D values over time was statistically significant, indicating a consistent and significant upward trend during 2020–2023 (Figure 1).

### 3.2 Subsystem-specific coordination analysis

**Figure 2:**
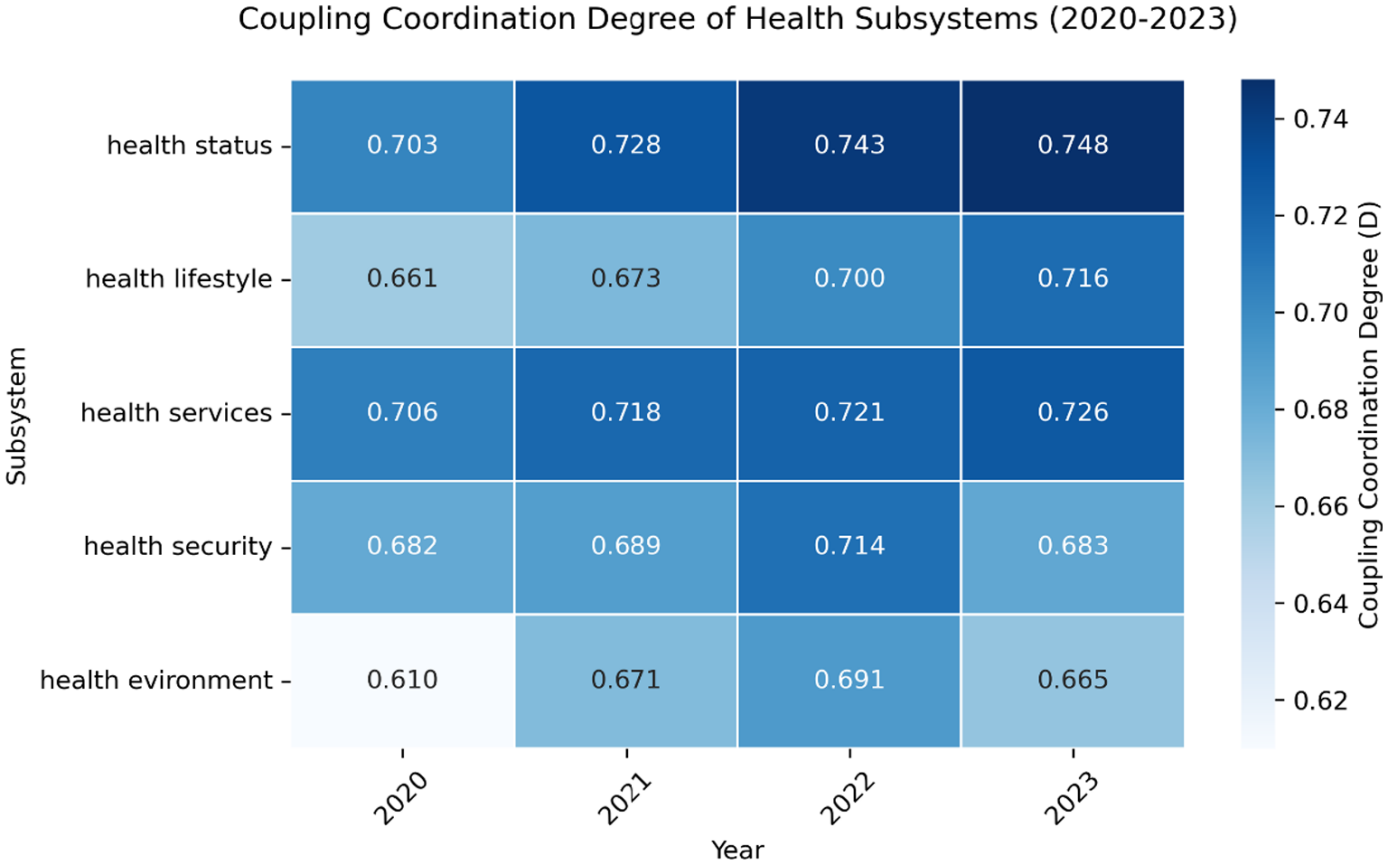
The results of the coupling and coordination degree between the five health subsystems and the economic and social development in Beijing.

The subsystem analysis reveals nuanced annual dynamics (Table 4; Figure 2).

**Table 4:** Coupling coordination degree of the subsystem situation in Beijing.

Subsystem	Year	C	T	D
Health status	2020	0.981	0.503	0.703
	2021	0.968	0.547	0.728
	2022	0.968	0.571	0.743
	2023	0.978	0.572	0.748
Health lifestyle	2020	0.997	0.439	0.661
	2021	0.995	0.455	0.673
	2022	0.991	0.494	0.700
	2023	0.992	0.517	0.716
Health services	2020	0.979	0.508	0.706
	2021	0.975	0.528	0.718
	2022	0.981	0.53	0.721
	2023	0.988	0.533	0.726
Health security	2020	0.991	0.469	0.682
	2021	0.99	0.479	0.689
	2022	0.985	0.517	0.714
	2023	0.999	0.466	0.683
Health environment	2020	0.996	0.373	0.610
	2021	0.996	0.452	0.671
	2022	0.994	0.48	0.691
	2023	1.000	0.443	0.665

#### 3.2.1 Health status

Maintained intermediate coordination throughout (D = 0.703–0.748). The C value dipped to 0.968 (−1.3%) during 2021–2022 before recovering to 0.978 (+1.0%) in 2023, while the T index grew consistently from 0.503 (2020) to 0.572 (+13.7%), peaking at 0.571–0.572 in 2022–2023.

#### 3.2.2 Health lifestyle

Transitioned from barely coordinated (D = 0.661, 2020) to primary coordination (D = 0.716, 2023). The C value showed a gradual 0.5% decline (0.997 → 0.992), contrasted by a 17.8% surge in T index (0.439 → 0.517), with the most significant annual T-index growth occurring in 2022 (+8.6%).

#### 3.2.3 Health services

Demonstrated exceptional stability, maintaining intermediate coordination (D = 0.706–0.726) with minimal C-value fluctuations (±0.6%). The T index increased steadily from 0.508 to 0.533 (+4.9%), though its growth rate halved from 3.9% (2020–2021) to 0.6% (2022–2023).

#### 3.2.4 Health security

Exhibited paradoxical trends – while C values improved from 0.991 to 0.999 (+0.8%), the T index plummeted 9.9% in 2023 (0.517 → 0.466), causing coordination regression from intermediate (D = 0.714, 2022) to primary level (D = 0.683, 2023).

#### 3.2.5 Health environment

Showed the most volatility, with D values oscillating between barely coordinated (0.610, 2020) and primary coordination (0.691, 2022). Despite achieving perfect C value (1.000) in 2023, a 7.7% T-index drop (0.48 → 0.443) reversed three-year coordination gains, returning to barely coordinated status (D = 0.665).

### 3.3 Spatiotemporal heterogeneity across districts

**Figure 3:**
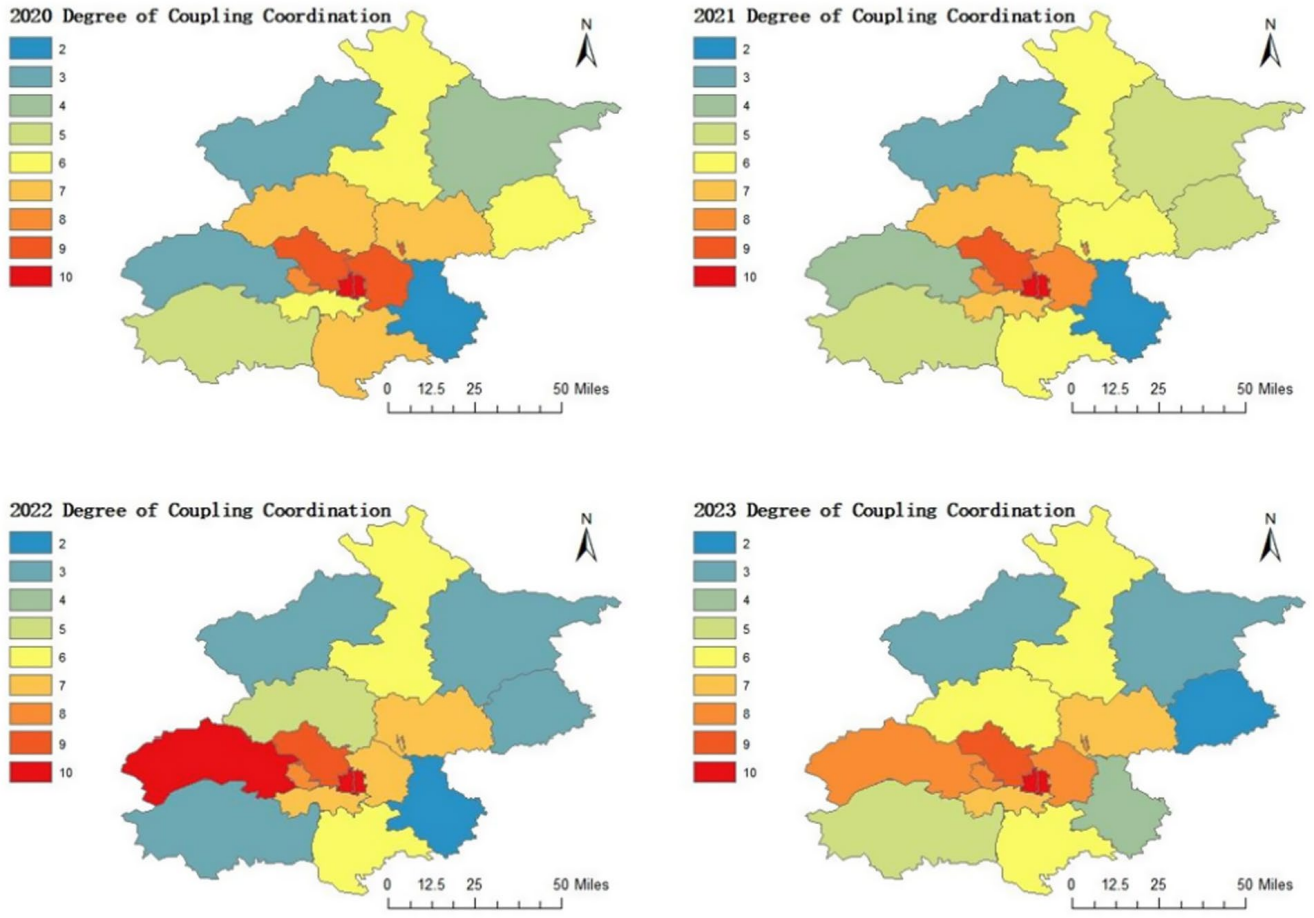
The results of the coupling and coordination degree between health and the economic and social development in 16 districts in Beijing.

Analysis of district-level coupling coordination reveals significant spatial disparities:

Core urban districts (e.g., Xicheng, Dongcheng) maintained premium coordination (D > 0.9) through integrated health-economic policies. Peri-urban districts (e.g., Chaoyang, Haidian) showed moderate progress but vulnerability to *T*-value fluctuations. Exurban districts (e.g., Pinggu, Yanqing) displayed chronic coordination deficits (D < 0.5), with environmental and infrastructure limitations perpetuating health-economic decoupling (Figure 3).

To quantify the spatial gradient of coupling coordination, Table 5 presents the complete statistical distribution of D-values across 16 districts in Beijing from 2020 to 2023, along with key quantitative disparity indicators (Coefficient of Variation [CV], Extreme Ratio [Max/Min], and Inter - Quartile Range [IQR]). The CV of district - level D-values decreased from 0.38 in 2020 to 0.32 in 2023, indicating a slight narrowing of spatial gaps. However, it remained above 0.3, reflecting persistent regional inequality.

**Table 5:** Statistical distribution of the coupling coordination degree (D-value) across 16 districts in Beijing (2020–2023).

Year	Mean D-value	SD	CV	Max/Min
2020	0.58	0.22	0.38	5.7 (0.92/0.16)
2021	0.61	0.23	0.38	5.5 (0.93/0.17)
2022	0.63	0.20	0.32	5.4 (0.94/0.17)
2023	0.65	0.21	0.32	5.3 (0.95/0.171)

#### 3.3.1 Urban synergy contrasts rural fragility

The Xicheng and Dongcheng districts maintained “Premium Coordination” status (D-value>0.9) for four consecutive years, with coupling degree (C) and coordination index (T) values approaching 1.0, indicating a high level of synergistic development in their socio - economic systems. Chaoyang and Haidian districts reached the “Good Coordination” tier (D-value 0.8–0.9), with Haidian demonstrating notable system resilience through continuous T-value improvement (0.793 → 0.745). In contrast, Tongzhou, Fangshan, and Pinggu Districts showed significant coordination imbalances. In 2023, Pinggu’s D-value dropped to 0.171, the lowest in the city. This was directly related to the changes in its concurrent health and economic indicators. According to the data in the file, its coupling degree C-value in 2023 was 0.612 and the coordination index T-value was 0.048, both at relatively low levels. From the perspective of the health system, its health resource - related indicators might have performed poorly, resulting in a low coordination index. From the economic system perspective, the low coupling degree might imply poor synergy between economic development and the health system. It is speculated that insufficient economic investment in supporting the health system might be the cause.

#### 3.3.2 Divergent development trajectories

While high-coordination districts demonstrated consistent performance (e.g., Dongcheng maintained D = 0.995 through 2023), differential developmental trajectories emerged across regions. Chaoyang experienced a transitional phase in 2022, with its coordination tier adjusting from Good to Primary (D = 0.691) alongside T-value variations (0.636 → 0.479). In Pinggu, system coupling metrics revealed complexity: while achieving perfect C = 1 in 2022, subsequent T-value reductions (to 0.076) introduced multidimensional equilibrium challenges requiring further investigation.

#### 3.3.3 Heterogeneous coordination improvement pathways

Districts manifested differential developmental pathways: Shijingshan maintained intermediate coordination levels throughout the study period, supported by stable coupling metrics (C = 0.937–0.986) and progressive T-value enhancement (0.59 → 0.631). Fengtai exhibited adaptive system behaviors, where moderate C-value adjustments (0.983 → 0.901) were counterbalanced by significant T-value growth (0.341 → 0.472), indicative of dynamic equilibrium maintenance between system coupling and coordination dimensions.

#### 3.3.4 Critical volatility requiring intervention

In 2023, Tongzhou District showed dynamic changes in its coordination characteristics. The coupling degree (C-value) decreased from 0.74 to 0.442, while the comprehensive development index (T-value) rose to 0.233 (maintaining “mild imbalance”). According to the data in the file, this “imbalanced improvement” has also been reflected in previous years. For example, in 2020, its coupling degree C-value was 0.575 and the coordination index T-value was 0.055. By 2023, although the T-value increased to some extent, the C-value decreased significantly. This change reflects that there may be an asynchrony between economic development and health system construction in Tongzhou District. It is possible that economic development has attracted resources such as population, but the construction of the health system has not kept pace in a timely manner, leading to a decrease in the coupling degree.

In 2022, Fangshan District showed dynamic changes in its indicators. The comprehensive development index (T-value) decreased from 0.241 to 0.101, and there was a slight change in coordination balance. According to the data in the file, its coupling degree C-value in 2022 was 0.879, which also changed to some extent compared with previous years. Combining with the previous trends, it is speculated that this change may be affected by external factors, such as environmental changes and policy adjustments, which have influenced the synergy between the health system and economic development, resulting in a decrease in the comprehensive development index.

#### 3.3.5 Distinct urban–rural coordination gradient

The analysis suggested a threefold “Core-Suburban-Exurban” differentiation pattern: Six core and suburban districts demonstrated relatively strong coordination (mean D = 0.873), whereas eight exurban districts showed notable gaps in this regard (mean D = 0.475). Yanqing District serves as an example of persistent coordination challenges, experiencing moderate imbalance (D < 0.3) over four consecutive years. During this period, its C-value and T-value followed diverging trends (C-value: 0.221 → 0.26; T-value: 0.404 → 0.291), which may indicate systemic gaps in the mechanisms for synchronizing urban and rural development.

### 3.4 Robustness test

To verify the stability of the CCD results, two robustness tests were conducted:

*Weight replacement test*: Replaced the entropy weight method with the AHP-Entropy combination weight method (AHP weight determined by expert scoring: health system = 0.5, economic system = 0.5; combination weight = 0.3 × AHP weight + 0.7 × Entropy weight). The correlation coefficient between the new CCD values and the original values was 0.92 (*p* < 0.01), indicating high consistency.

*Indicator deletion test*: The “per capita GDP” (an economic indicator) was excluded, and three core coupling coordination indicators—Coupling Degree C, Comprehensive Coordination Index T, and Coupling Coordination Degree D—were recalculated. Subsequent ordinary least squares (OLS) regression (with post-deletion data as the dependent variable and pre-deletion data as the independent variable) yielded an R-squared/Adjusted R-squared of 1.000 for all three indicators, a main variable coefficient of 1.0000, large F-statistics, and extremely small *p*-values (all < 0.001). These results confirm the model’s significance. This further demonstrates that the coupling coordination results are relatively robust to the exclusion of “per capita GDP,” though Coupling Degree C exhibits higher sensitivity. The two tests showed that the CCD results of this study were stable and reliable.

## 4 Discussion

The findings of this study provide valuable insights into the coupling and coordination relationship between the construction of “Healthy Beijing” and economic development. The results indicate that from 2020 to 2023, the overall coupling coordination degree between Beijing’s health system and socio-economic development showed a steady upward trend, transitioning from “basic coordination” to “intermediate coordination.” This reflects the positive interaction and mutual reinforcement between health and economic development in Beijing, driven by policy initiatives such as the “Healthy China” strategy and investments in public health infrastructure. However, the analysis also reveals significant disparities in coordination levels across sub-systems and districts, highlighting areas requiring further attention and intervention.

### 4.1 Overall coupling coordination trends

The progressive improvement in the coupling coordination degree (D value) from 0.68 in 2020 to 0.714 in 2023 demonstrates that Beijing’s health initiatives have effectively aligned with economic development goals. This aligns with global trends where health and economic systems are increasingly recognized as interdependent factors influencing societal well-being (30, 31). The steady increase in coordination levels can be attributed to Beijing’s comprehensive health policies, such as enhanced medical infrastructure, improved service quality, and health education campaigns, which have not only elevated public health awareness but also stimulated demand for health-related services, thereby contributing to economic growth (32, 33).

### 4.2 Sub-system coordination analysis

The five health sub-systems exhibited varying degrees of coordination with socio-economic development. Health level and health services consistently maintained “intermediate coordination,” reflecting the effectiveness of Beijing’s public health infrastructure and service improvements. The health lifestyle sub-system showed notable progress, transitioning to “intermediate coordination” in 2023, likely driven by policies promoting healthy behaviors such as fitness initiatives and public health campaigns (34, 35). However, the health security and health environment sub-systems lagged, with health security experiencing significant fluctuations and health environment remaining in “basic coordination.” These findings suggest that while Beijing has made strides in certain health domains, resource allocation and policy implementation in areas like environmental governance require urgent attention to ensure balanced development.

### 4.3 District-level disparities

The analysis of Beijing’s 16 districts revealed a pronounced “core–suburban–exurban” gradient in coupling coordination levels, a pattern rooted in Beijing’s unique urban governance logic, fiscal allocation mechanism, and population mobility dynamics—rather than mere “socio-economic resource gaps.” From the perspective of urban development theory (Central-Periphery Model), this gradient reflects the unbalanced spatial spillover of public health resources under Beijing’s “strong core” spatial structure; from the public finance theory (Tiebout Model), it stems from the mismatch between local fiscal capacity and public health demand.

This spatial disparity is closely linked to fiscal expenditure allocation and population pressure. From the perspective of fiscal expenditure, differences in the orientation of fiscal transfer payments and fund allocation efficiency across districts exert an impact on the supply of public health resources. In terms of population pressure, changes in population structure and mobility patterns further widen the coordination gap between the promotion of the Healthy Beijing initiative and economic development. Due to data privacy protection requirements, the specific data involved in this section is not publicly disclosed, and this adjustment does not affect the core conclusions of the paper.

Population pressure: Population structure changes and policy-driven population mobility further widen the coordination gap. From the demographic data, exurban districts have dual pressure of ‘aging + population outflow’: Yanqing’s 65 + population ratio reached 17.49% in 2023 (close to Dongcheng’s 20.63%), but 38% of working-age population (15–64 years) moved to core/suburban districts for employment, resulting in a ‘low fiscal revenue + high health demand’ dilemma. In contrast, core districts (e.g., Xicheng) attract high-income and high-human-capital groups, with 92.31% of Grade II + public hospitals having geriatric departments (vs. 66.67% in Pinggu) and 13.37 licensed physicians per 1,000 population (vs. 4.44 in Pinggu)—effectively matching the aging population’s needs. Notably, Tongzhou, as Beijing’s sub-center, experienced an 8.5% permanent population growth (2020–2023) due to the ‘relocation of non-capital functions,’ but its licensed physicians per 1,000 population only increased from 2.44 to 2.77, leading to a decoupling between population inflow and health service supply.

### 4.4 Policy implications

The study highlights the need for a more integrated approach to health and economic development. Guided by the Central-Periphery Model and fiscal equalization theory, targeted policies should address the root causes of spatial disparities:

Optimize the fiscal transfer payment formula for public health: Incorporate “health resource gap” and “aging pressure” into the transfer payment calculation (e.g., increasing the weight of 65 + population ratio and physician density gap), ensuring that exurban districts receive at least 25% of health transfer payments—closing the absolute gap in health expenditure between core and exurban districts within 3 years.

Align health resource allocation with population and policy dynamics: For Tongzhou (sub-center), link health facility construction to population growth (e.g., adding 1 licensed physician per 10,000 new residents); for exurban districts (e.g., Pinggu), leverage the “coordinated development of Beijing-Tianjin-Hebei” policy to attract medical talents through cross-regional talent subsidies.

Establish a “health-policy feedback mechanism”: Embed health coordination indicators (e.g., district-level D-value) into the performance evaluation of municipal and district governments, ensuring that policies such as “Relieving Beijing of functions non-essential to its role as the capital” are accompanied by synchronous health resource relocation (e.g., building branch hospitals of core district hospitals in exurban areas). Additionally, addressing the volatility in health security and the lag in health environment coordination demands long-term strategic planning and cross-sector collaboration to ensure sustainable development.

### 4.5 Limitations and future research

While this study provides a comprehensive evaluation of the coupling coordination relationship, several limitations warrant mention. First, the study uses only 4 years of data (2020–2023), which limits the identification of long-term trends. Second, due to data availability, this study only conducts quantitative analysis and lacks qualitative research, which may lead to incomplete interpretation of spatial disparities. Third, the spatial analysis does not consider the influence of commuting flows, which may underestimate the actual health service accessibility of exurban districts. Future research should address these limitations by extending the data period and integrating qualitative methods. Second, the study focuses on quantitative indicators, but qualitative analyses of policy effectiveness and public perception could offer deeper insights. Finally, incorporating dynamic factors such as technological advancements and demographic shifts could enhance the robustness of future models.

## Data Availability

The original contributions presented in the study are included in the article/supplementary material, further inquiries can be directed to the corresponding author.
