# Correction: Evaluating the coupling coordination between Healthy Beijing initiative and economic development

**DOI:** 10.3389/fpubh.2026.1797876

**Published:** 2026-02-16

**Authors:** Xi Wang, Yueying Cui, Jiu Cheng, Mingming Gao, Yifei Wang, Ruihua Feng

**Affiliations:** 1 Institute of Medical Information / Medical Library, Chinese Academy of Medical Sciences and Peking Union Medical College, Beijing, China; 2 Union Hospital, Tongji Medical College, Huazhong University of Science and Technology, Wuhan, China

**Keywords:** Healthy Beijing initiative, coupling coordination, Healthy China initiative, economic development, evaluating

A correction has been made to section **4 Discussion**, subsection *4.3 District-level disparities*, paragraphs 2 and 3, due to privacy concerns related to specific data included in the original text.

The paragraphs were erroneously included with privacy-related specific data, which does not meet data privacy protection requirements.

The corrected paragraph, which maintains contextual fluency and complies with data privacy protection norms, is as follows:

“This spatial disparity is closely linked to fiscal expenditure allocation and population pressure. From the perspective of fiscal expenditure, differences in the orientation of fiscal transfer payments and fund allocation efficiency across districts exert an impact on the supply of public health resources. In terms of population pressure, changes in population structure and mobility patterns further widen the coordination gap between the promotion of the Healthy Beijing initiative and economic development. Due to data privacy protection requirements, the specific data involved in this section is not publicly disclosed, and this adjustment does not affect the core conclusions of the paper.”

The original version of this article has been updated.

